# The ER Stress Induced in Human Neuroblastoma Cells Can Be Reverted by Lumacaftor, a CFTR Corrector

**DOI:** 10.3390/cimb46090553

**Published:** 2024-08-24

**Authors:** Michela Pecoraro, Adele Serra, Maria Pascale, Silvia Franceschelli

**Affiliations:** 1 Department of Pharmacy, University of Salerno, Via Giovanni Paolo II, 84084 Fisciano, Salerno, Italy; mipecoraro@unisa.it (M.P.); adserra@unisa.it (A.S.); pascale@unisa.it (M.P.); 2 Ph.D. Program in Drug Discovery and Development, University of Salerno, 84084 Fisciano, Salerno, Italy

**Keywords:** neuroblastoma cells, ER stress, oxidative stress, protein misfolding, Lumacaftor

## Abstract

Most neurodegenerative diseases share a common etiopathogenesis, the accumulation of protein aggregates. An imbalance in homeostasis brought on by the buildup of misfolded proteins within the endoplasmic reticulum (ER) results in ER stress in the cell. Three distinct proteins found in the ER membrane—IRE1α, PERK, and ATF6—control the unfolded protein response (UPR), a signal transduction pathway that is triggered to restore normal physiological conditions. Buildup of misfolded proteins in ER lumen leads to a shunting of GRP78/BiP, thus triggering the UPR. PERK autophosphorylation leads to activation of ATF4, the transcription factor; finally, ATF6 activates the UPR’s target genes, including GRP78/Bip. Accordingly, the UPR is a cellular reaction to an ER stress state that, if left unchecked for an extended period, results in apoptosis and irreversible damage. The identification of caspase 4, which is in the ER and is selectively activated by apoptotic stimuli caused by reticular stress, further demonstrated the connection between reticular stress and programed cell death. Moreover, oxidative stress and ER stress are linked. Oxidative stress is brought on by elevated quantities of radical oxygen species, both mitochondrial and cytosolic, that are not under the enzymatic regulation of superoxide dismutases, whose levels fall with increasing stress. Here, we evaluated the activity of Vx-809 (Lumacaftor), a drug used in cystic fibrosis, in SH-SY5Y neuronal cells, in which an ER stress condition was induced by Thapsigargin, to verify whether the drug could improve protein folding, suggesting its possible therapeutic use in proteinopathies, such as neurodegenerative diseases (NDs). Our data show that Vx-809 is involved in the significant reduction in protein produced under ER stress, particularly in the levels of Bip, ATF4, and ATF6 by Western blotting analysis, the reduction in ROS in the cytosol and mitochondria, and the reduction in the activation of the apoptotic pathway, measured by flow cytofluorimetry analysis and in restoring calcium homeostasis.

## 1. Introduction

NDs are characterized by a progressive loss of neuronal function in specific regions of the nervous system, culminating in severe dysfunction [1]. NDs include highly debilitating illnesses, such as Parkinson’s disease (PD), characterized by ubiquitinated protein deposits called Lewy bodies (composed of α-synuclein) in the neuronal cytosol; Alzheimer’s disease (AD) in which intracellular deposits of tau are observed in neurofibrillary tangles and in which extracellular aggregates of amyloid β are seen in senile plaques; Huntington’s disease, due to an aggregate of mutant huntingtin proteins and prion diseases (PrDs), are well known to form protein aggregates and are causally involved in the pathogenesis of a group of transmissible spongiform encephalopathies, including Creutzfeldt–Jakob disease and kuru [1]. Despite significant variations in clinical presentation and prevalence, NDs share several characteristics, such as their chronic and progressive nature, age-related increase in prevalence, degeneration of neurons in particular regions of brain, damage to synaptic networks, and selective damage of brain tissue. NDs are also characterized by aggregation of intrinsically disordered proteins (IDPs) (also called natively unfolded proteins), which refers to proteins that do not have an independently folded state. In many circumstances, interactions between intrinsically disordered proteins and target proteins or nucleic acids result in folding of the binding domain; in other cases, binding causes merely local ordering of short, extended, or helical portions of polypeptides [2].

Proteins are chains of amino acids consisting of 20 different L-α-amino acids, which fold into unique three-dimensional structures. The form in which a protein naturally folds is called its ‘native state’, which is determined by its amino acid sequence. Their three-dimensional structure contributes greatly to the performance of biological processes by cells.

Even though the protein aggregates involved in distinct NDs are different, the process of protein misfolding, its intermediates, end-products, and main features are remarkably similar [3].

Neuronal cells are very sensitive to protein misfolding, therefore excessive misfolding and aggregation results in synaptic dysfunction, apoptosis, and selective neuronal death [4,5].

Misfolded proteins can have two harmful effects: either they cause a deleterious “gain of function”, as seen in many neurodegenerative diseases like AD, PD, and HD, where the formation of harmful amyloid is the result of protein misfolding, or they cause loss of function, as seen in cystic fibrosis (CF) and α1-antitrypsin deficiency. In other instances, on the other hand, the changes are quite small, and the resultant proteins only exhibit a slight reduction in their typical activity [3]. Susan Lindquist, a prion researcher, states that “up to half of all human diseases could be caused by protein misfolding”.

Since protein homeostasis stops aberrant protein aggregation, it is essential for maintaining cellular health and function. Under normal conditions, misfolded proteins are recognized and made more easily degradable by the proteasome, lysosome, and autophagy pathways, whereas chaperones found in the cytosol and endoplasmic reticulum (ER) guarantee clear folding of freshly generated native proteins [6].

More than one-third of all proteins produced by a cell are synthesized, folded, and structurally matured in the ER. In addition, the ER coordinates many cellular processes, such as the maintenance of intracellular calcium levels, synthesis of lipids, and the folding and processing of most polypeptides intended for secretion [7].

Proteins must be folded into their distinct three-dimensional structures and undergo various post-translational processes, such as N-glycosylation and disulfide bonds formation, in the ER lumen [8].

Degenerative disorders are caused by protein aggregation in neurons that stop functioning properly. Protein aggregation and accumulation of misfolded protein cause ER stress, which activates the unfolded protein response (UPR), a protection mechanism that, through several transcriptional and translational modifications, acts both to increase the folding capacity of the ER and to decrease the load of unfolded proteins. Protein kinase RNA (PKR)-like ER kinase (PERK), inositol-requiring enzyme 1 (IRE1), and activating transcription factor 6 (ATF6) are the three transmembrane ER proteins that constitute the UPR. Under resting conditions, all three proteins form a complex with the chaperone GRP78/BiP. In response to an accumulation of misfolded proteins in the ER, BiP dissociates and binds the misfolded proteins. PERK, IRE1, and ATF6 are activated when BiP dissociates [9,10].

In short, after detection of unfolded proteins, IRE1 is activated and triggers the regulated splicing of an unconventional 26 nt intron of X-box binding protein 1 (Xbp1). This results in a frameshift, producing the spliced Xbp1 isoform (XBP1s), which acts as a transcription factor [7]. PERK is a protein kinase that reacts to extracellular reticulum stress by homo-multimerization and autophosphorylation. When PERK is activated, it phosphorylates the α subunit of eIF2α, which inhibits translation and stops the cytoplasm from synthesizing new proteins. Nevertheless, the activation of transcription factor 4 (ATF4), which in turn stimulates the transcription of UPR and apoptotic genes, including the pro-apoptotic C/EBP protein homolog (CHOP), is preferentially induced by phospho-eIF2α [9,11].

ATF6 travels from the ER to the Golgi apparatus in response to ER stress, where it is cleaved, moved to the nucleus, and stimulates the production of Xbp-1 and genes needed for ER-associated protein degradation (ERAD). Proteins that are terminally unfolded or misfolded are taken out of the ER by ERAD so that proteasomes can break them down [12].

The precise association between misfolded proteins and the etiology of neurodegenerative disorders is still unknown. Moreover, there is ongoing debate on the potential essential role of endogenous stress in neurodegenerative disorders.

Neurons can be killed by the accumulation of toxic protein species, and increasing research indicates that ER stress is a key mechanism causing this neurotoxicity [5]. In postmortem brain and spinal cord tissues from various NDs, activation of IRE1α and induction of UPR have been identified [13,14]. Several scientists attempted to alter the UPR murine neurodegenerative models in vivo in light of this evidence. In prion-infected mice, for instance, Moreno and associates [15] discovered that oral treatment of a highly selective PERK inhibitor that successfully penetrates the blood–brain barrier dramatically reduced neurodegeneration and clinical illness. Given the strong evidence of UPR activation in human patients, studies like these are raising awareness of the possibility that pharmacological modulation of the UPR may have disease-modifying advantages for a variety of neurodegenerative illnesses. Since calcium is an essential component of many intracellular processes and serves as a second messenger, the cytoplasmic calcium is actively transported out of cells and stored in the ER or mitochondria, hence maintaining very low concentrations of the mineral. For instance, dysregulation of cytosolic Ca^2+^ is seen in AD, which leads to ER stress-mediated apoptosis and ER malfunction. Moreover, an elevated Ca^2+^ influx that results in cell death is also seen in PD [16,17]. The redox balance in the ER is related to both protein folding and cellular calcium homeostasis. In the ER, oxidation is also used in an oxidative protein folding process to promote disulfide bond formation. Chronic ER stress-related neurodegeneration can be caused by a synergistic combination of protein misfolding and reactive oxygen species (ROS) accumulation [18]. Common issues in several NDs include redox status, ER stress, and protein misfolding that results in neurodegeneration. Even though the molecular mechanisms underlying NDs are well understood, effective treatments or a cure is yet unknown for these conditions. Therapeutic intervention focuses mostly on misfolded protein aggregates. Several approaches are under development to prevent the formation of or to remove misfolded aggregates.

Protein misfolding-induced neurotoxicity can be inhibited by increased chaperone expression, indicating a potential utility for chaperones as therapeutic agents. Chaperones, whether natural, synthetic, or pharmaceutical, have demonstrated promise as therapeutics for the management of numerous protein conformational diseases [19].

Lumacaftor (Vx-809) is a CFTR corrector, and it was the second treatment to be approved for use in CF therapy, though it can only be used in conjunction with Ivacaftor, a potentiator that improves channel function; also, it has been shown to be safe with biological activity in a Phase IIa clinical trial [19]. The drug has been shown in vitro to correct p. Phe508del misprocessing of CFTR and increase the amount of protein localized on the cell surface [20]. This compound is based on a di-fluorobenzodioxolyl-cyclopropane linked to a substituted arylpyridine via an amide bond discovered by screening compounds that enhance Phe-508del-CFTR-mediated chloride transport [21].

Its mechanism of action and their binding sites are still incompletely understood, but several studies indicate that it may stabilize folding of the molecule through direct binding to the first nucleotide-binding domain (NBD1) or promoting interactions between the first cytoplasmic loop (CL1) of transmembrane domain 1 (TMD1) and NBD1 [21,22]. Furthermore, it is not CFTR-specific because it was demonstrated to stop the trafficking of another defective ABC transporter (ABCA4), which has significant similarity with CFTR NBDs [9].

The approval of Lumacaftor in therapy represents the genesis of a new era of medicine in the treatment of CF, with a positive impact on patients’ lives [19,21].

In previous work Pecoraro and co-workers [9] demonstrated how the Vx-809 corrector counteracts the activation of the UPR pathway by acting on protein folding on adenocarcinomic human alveolar basal epithelial (A549) and malignant melanoma (A375) cells.

**Figure 1:**
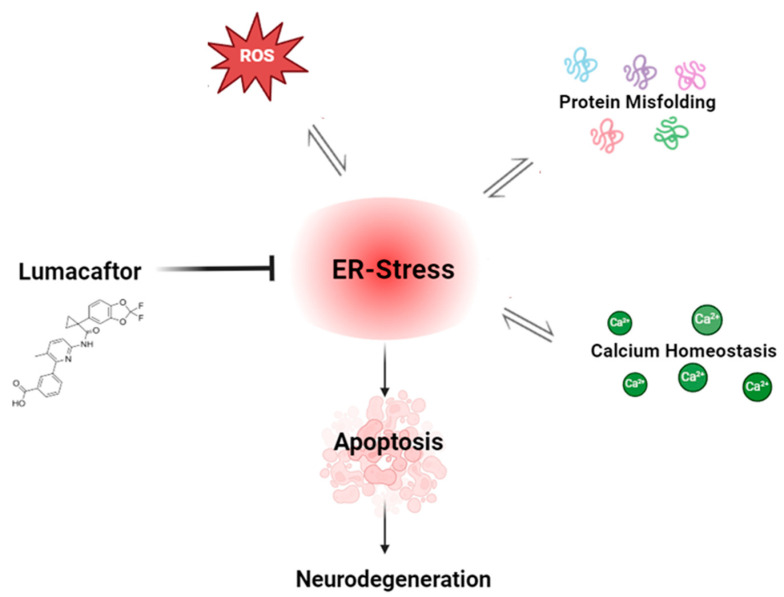
Schematic representation on the probable effect of Lumacaftor.

Therefore, in this study, we investigated if the off-label mechanism of Vx-809 is also confirmed in a neuroblastoma cell model, often used as in vitro models of neuronal function and differentiation, leading to the hypothesis that the drug interacts not only with the CFTR protein but, by an unknown mechanism, also on other misfolded proteins, demonstrating that it can also be used in other diseases, like NDs (Figure 1).

## 2. Materials and Methods

### 2.1. Reagents

Vx-809 (S1565) was acquired from Selleckchem (Houston, TX, USA). The monoclonal antibodies used included the following: anti-Grp78/BiP (3177, Cell Signaling, Danvers, MA, USA), anti-ATF6 (658805, Cell Signaling, Danvers, MA, USA), anti-ATF4 (10835-1-AP, Proteintech, San Diego, CA, USA), anti-PERK (3192, Cell Signaling), anti-MnSODIII (sc-271170), anti-actin (TA811000, OriGene, Rockville, MD, USA), anti-tubulin (sc-5286), anti-caspase 4 (sc-1229). Secondary antibodies (anti-rabbit, A120-101P, and anti-mouse, A90-137P) were bought from Bethyl Laboratories (Montgomery, TX, USA). The Texas red-conjugated secondary antibody (T6390 and PA1-28662) was procured from Thermo Fisher Scientific (Waltham, MA, USA). The mitochondrial superoxide indicator MitoSOX Red (M36008) was purchased from Invitrogen, ThermoFisher (Waltham, MA, USA). Thia-zolyl blue tetrazolium bromide, H_2_DCF-DA, Thapsigargin, propidium iodide, FURA 2AM, and ionomycin were all purchased from Sigma-Aldrich (St. Louis, MO, USA).

### 2.2. Cell Culture

A human neuroblastoma cell line (SH-SY5Y, CL0208, Elabscience, Houston, TX, USA) was subcultured weekly in 75 cm^2^ sterile culture flasks with Dulbecco’s modified Eagle’s Medium/F12 (DMEM/F12; Euroclone, Pero (Milan), Italy) containing 10% fetal bovine serum (FBS; Euroclone), 100 U/mL penicillin, and 100 μg/mL streptomycin in a humidified atmosphere of 5% CO_2_ at 37 °C. The experiments were always performed at a confluence of less than 80% of the cells.

### 2.3. Experimental Protocol

The cells were seeded and, after 24 h of adhesion, were pretreated with 300 nM Thapsigargin (TG) for 30 min, 1 h, and 4 h, to induce an ER stress condition. Then, after the TG was removed, fresh media, with and without Vx, were added to cells. Corrector Vx-809 (2 µM) was administered for 24 h, and then, cell lysates were collected after 24 h of treatment for both the Vx- and TG-treated cells.

### 2.4. Cell Viability

The potential cytotoxic effects of Vx-809 and TG on neuroblastoma cells were investigated by MTT (3-[4,5-dimetiltiazol-2,5-diphenyl-2H-tetrazolium bromide]) assay, as reported in Pecoraro et al. 2018 [23]. In summary, cells (4.5 × 10^3^/well) were seeded in 96-well plates, and after 24 h, they were treated as described above. Staurosporin (Sp) 0.2 µM was used as a positive control.

At the end of treatment, 25 μL of MTT (5 mg/mL) was added to each well, and the plates were incubated for other 1 h to allow the formation of formazan salt. After this, salt formazan was solubilized with 100 μL of a solution containing 20% (*w*/*v*) SDS, 50% (*v*/*v*) *N*, *N*-dimethylformamide, at pH 4.5. The absorbance at 620 nm was analyzed by a Multiskan Spectrum Thermo Electron Corporation Reader. Cell vitality was calculated as % vitality = 100 × (OD_treated_/OD_DMSO_).

### 2.5. Protein Extraction and Western Blotting Assay

The cells (1 × 10^6^ cells/plate) were plated and processed as described in the experimental procedure. Proteins were extracted from the cells by incubation with lysis buffer (20 mM Tris-HCl pH 7.5, 1 mM EGTA, 150 mM NaCl, 1 mM Na_2_EDTA, 1% sodium deoxycholate, 1× protease and phosphatase inhibitor cocktail, and 2% IGEPAL), for 30 min. Afterwards, the lysates were centrifuged at 14,000 rpm for 15 min at 4 °C. Protein content was determined by the Bradford protocol, and 60 µg of protein was loaded onto 8–10% acrylamide gels and separated by SDS-PAGE, in denaturing conditions, and transferred to nitrocellulose membranes by means of a minigel apparatus (Bio-Rad Laboratories, Richmond, BC, Canada). Next, the blots were blocked in 5% Tris-buffered saline in non-fat dry milk for 2 h at room temperature and treated with primary antibodies anti-BiP or anti-ATF4 or ATF6 or anti-PERK or anti-SODIII for an entire night at 4 °C. Actin or tubulin was used as loading control. After various washes with PBS/0.1% Tween, the appropriate secondary antibodies were also added for 1 h at room temperature. The immunoreactive protein bands were viewed with enhanced chemiluminescence (ECL) reagents and blot imaging (LAS 4000; GE Healthcare, Milan, Italy). Western blotting imaging data were quantified using ImageJ software Version 1.54j [9,20].

### 2.6. RNA Extraction and Real-Time RT-PCR Protocol

SH-SY5Y cells were seeded in 100 mm culture dishes and treated as above. RevertAid First Strand cDNA Synthesis Kit (K1622) was used to retrotranscribe 0.5 µg of total RNA that was extracted using TRI Reagent (T9424, Sigma-Aldrich, Darmstadt, Germany) in accordance with the manufacturer’s instructions. Two microliters of cDNA were subjected to semi-quantitative PCR using the subsequent primers: 5′-C CTT CTG GGT AGA CCT CTG GGA G-3′ and 5′-A AAC AGA GTA GCA GCT CAG ACT GC-3′. By staining 4% agarose gels with ethidium bromide and subjecting them to LAS 4000 (GE Healthcare, Chicago, IL, USA), fragments of 452 and 426 bps, which correspond to un-spliced and spliced XBP1 mRNA, were found.

### 2.7. Calcium Signaling Assay

Intracellular calcium concentrations were measured using a membrane-permeant acetoxymethyl ester form of Fura 2, the Fura 2-AM, a fluorescent indicator dye. Neuroblastoma cells (3 × 10^4^ cells/plate) were seeded in 6-well tissue culture plates and treated as previously described.

At the final stage of the treatment, the cells were rinsed with phosphate-buffered saline (PBS), reconstituted, and incubated for 45 min in 1 mL of Hank’s balanced salt solution (HBSS) that contained 5 μM Fura 2-AM.

After removing excess Fura 2-AM, SH-SY5Y cells were transferred to a cuvette for spectrofluorometer measurement (Per-kin-Elmer LS-55, Milano (MI), Italy). The cells were then rinsed with PBS buffer. The appropriate quantities of TG (1 nM) and Ionomycin (1 μM) were added to the cuvette in calcium-free HBSS/0.5 mM EGTA buffer to initiate treatment. At 515 nm, emission fluorescence was seen, with the excitation wavelength being varied between 340 and 380 nm. As previously reported [24], the fluorescence intensity ratio of 340/380 nm (F340/F380) was strongly correlated with intracellular free calcium. The TG or Ionomycin-induced increases in the fluorescence ratio (F340/F380 nm) were measured and reported as a delta (Δ) increase in the fluorescence ratio (F340/F380 nm).

### 2.8. Intracellular and Mitochondrial ROS Detection

Cytosolic ROS generation was evaluated in neuroblastoma cells (8 × 10^4^ cells/well) plated into 6-well plates and treated as depicted above. After the treatment, the cells were collected, washed twice with PBS, and then incubated in PBS containing 2′,7′-dichlorofluorescin diacetate (H_2_DCF-DA; 10 μM) probe, at 37 °C for 30 min in the dark. Cell fluorescence was then measured using fluorescence-activated cell sorting (FACSscan; Becton–Dickinson) and analyzed with Cell Quest software.

Changes in mitochondrial O_2_^−^ production were evaluated by fluorescent mitochondrial superoxide indicators (MitoSOX Red). Briefly, cells (8 × 10^4^ cells/well) were incubated with MitoSOX Red (2.5 μM) for 15 min in the dark at 37 °C, followed by washout. Fluorogenic dye, MitoSOX Red, is used to detect superoxide very specifically in living cell mitochondria. Once in mitochondria, it is oxidized by superoxide and produces red fluorescence, which is measured by flow cytometry (FACScan; BD Biosciences, San Jose, CA, USA) and analyzed by Cell Quest software version 4.1.

### 2.9. Caspase 4 Measurement

Caspase 4 cleavage was analyzed by fluorescence-activated cell sorting (FACSscan; Becton–Dickinson). SH-SY5Y cells were cultured in a 24-well plate (8 × 10^4^ cells/well) and, after 24 h of adhesion, were treated as previously described. Subsequently, the cells were collected and treated for 20 min with a fixing solution (2% FBS, 4% formaldehyde, and sodium azide 0.1% in PBS), permeabilized with a solution consisting of 4% formaldehyde, Triton X-0.1%, 2% FBS, 0.1% sodium azide, and PBS, and the samples were left for 30 min. Following this, anti-caspase 4 antibody was added, and a secondary antibody of goat Texas-Red was utilized. After washing, the cells were fixed and detected by flow cytofluorometry and analyzed by Cell Quest software. The data were represented as the percentage of positive cells.

### 2.10. Hypodiploid DNA Detection

The cells were plated (8 × 10^4^ cells/well) in a 24-well plate and allowed to grow for 24 h and treated as described above. After two PBS washes, the fixed cells were resuspended in 500 μL of 0.1% sodium citrate buffer, 0.1% Triton X-100, and 50 μg/mL of propidium iodide (PI). The cells were then incubated for 30 min at 4 °C in the dark to analyze the DNA content. By applying Cell Quest software, flow cytofluorometry was used to analyze PI-stained cells. The study of cellular debris was stopped by raising the scatter threshold, and the logarithmic scale was used to record the amount of DNA in the nucleus. The percentage of the hypodiploid region is used to represent the results.

### 2.11. Analytical Statistics

The commercially available software GraphPad Prism8 (GraphPad Software Inc., San Diego, CA, USA) was used for data assessments and statistical analysis. The data are shown as the mean ± standard error of at least three distinct experiments that were carried out in technical triplicate. Statistical information was gathered using the non-parametric Mann–Whitney U technique between the experimental points. If the *p* values fell between <0.01 and 0.05, the differences were regarded as significant.

## 3. Results

### 3.1. Role of Vx-809 in UPR Pathway

TG mimics the ER stress condition by blocking the sarco/endoplasmic reticulum Ca^2+^-ATPase (SERCA), disrupts Ca^2+^ homeostasis, and causes cell death [25]. Considering the above considerations, by using the MTT assay on neuroblastoma cells to evaluate the potential toxic effect and in our experimental model, using 300 nM TG, we observed cell viability higher than 50%.

ER homeostasis plays a vital role in cell physiological functions. Several factors can destroy ER homeostasis, triggering a stress condition which activates an UPR pathway. Vx-809 involvement on misfolding protein was evaluated by a Western blotting assay on cell lysates, analyzing some UPR pathway proteins after inducing ER stress in human neuroblastoma (SH-SY5Y) cells.

One of the most essential protective mechanisms induced by the UPR is the upregulation of the GRP78/BiP expression [26].

Western blotting analysis on SH-SY5Y extracts revealed that TG treatment significantly increased (*p* < 0.001) Grp78/BiP levels at all pretreatment times, confirming an ER stress condition.

**Figure 2:**
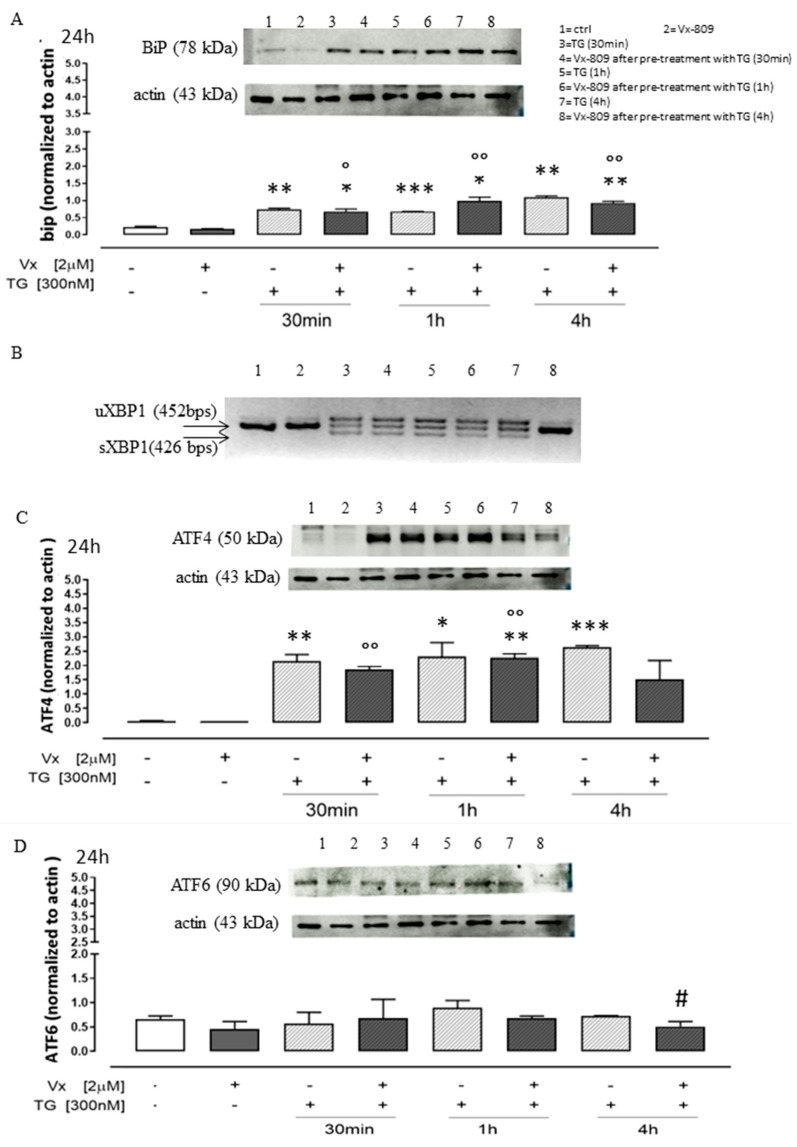

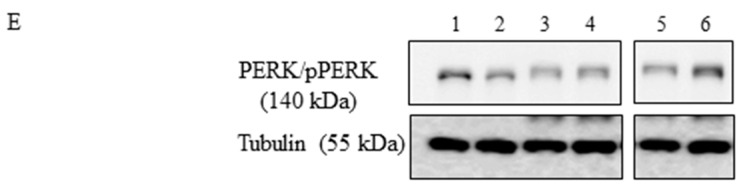
Vx-809 affects UPR activation. Cells were pretreated with 300 nM TG for 30 min or 2 or 4 h, to induce ER stress condition. Next, Vx-809 (2 µM) was added for 24 h. BiP (Panel (**A**)), ATF4 (Panel (**C**)), ATF6 (Panel (**D**)) and PERK/pPERK (Panel (**E**)) expressions in neuroblastoma cells were revealed by Western blotting analysis. Expression of actin or tubulin was employed as a loading control. Panel (**B**) illustrates the gel migration of the 452 bps unsplit fragment of XBP1 mRNA (XBP1 u) and the 426 bp split fragment (XBP1 s), achieved by RT-PCR. These results are presented as the average ± standard error of at least three separate, triplicate-performed studies. Data were processed according to the Mann–Whitney U-test. * *p* < 0.05, ** *p* < 0.005, and *** *p* < 0.001 vs. untreated cells; ° *p* < 0.05 and °° *p* < 0.005 vs. Vx-809-treated cells; # *p* < 0.05 vs. TG-treated cells.

Addition of Vx-809 reduced TG-induced Grp78/BiP overexpression, significantly (*p* < 0.05) at 30 min and 4 h (*p* < 0.005) pretreatment, as shown in Figure 2A. Moreover, RT-PCR experiments showed the corrective Vx-809 action on splicing of XBP1 (Figure 2B), mediated by IRE1 activation.

ER stress is a known inducer of ATF4 protein expression [27]. Accordingly, we tested the Vx-809 effect on ATF4 induction in response to chemically mediated ER stress. As expected, ATF4 protein was significantly induced (*p* < 0.001) at all time points in our experimental model, following TG treatment. Corrector Vx-809 treatment significantly reduced (*p* < 0.005) TG-induced ATF4 levels, confirming its effect on ATF4 activation in neuroblastoma cells (Figure 2C).

In mammalian cells ATF6 is an n-type membrane protein located in the ER. Several studies have shown that increased ATF6 expression regulates ER stress, reducing cellular damage and ultimately exerts a neuroprotective effect. Previous research has demonstrated that ATF6 deficiency enhances organ damage, as proven by the more severe functional damage and poorer prognosis exhibited in ATF6 knockout mice following cerebral ischemia. More current works have demonstrated that improving brain function is facilitated by activating the ATF6 signaling pathway in the brain [28,29,30]. The results obtained on neuroblastoma cells, by Western blotting analysis, showed that total ATF6 expression increased in cells pretreated at 1 h and 4 h with TG compared with control cells, confirming the induction of reticular stress and the onset of UPR. Vx-809 administration significantly reduced (*p* < 0.05) TG-induced total ATF6 levels, in particular at 4 h TG-pretreatment times (Figure 2D), restoring a physiological condition.

The PERK pathway is a crucial mediator in neurodegeneration observed in several protein misfolded diseases, suggesting that the general disruption of protein translation is the mechanism underlying neuronal dysfunction and degeneration. In a PD case, markers of PERK activation were found in PD postmortem tissues, where nigral dopaminergic neurons showing αSyn inclusion were also positive for phosphorylated PERK [31]. Therefore, using the SH-SY5Y cell line, we performed a Western blotting analysis of the phosphorylated form of PERK (p-PERK). First and foremost, immunoblots were used to identify PERK phosphorylation. Figure 2E illustrates this process and shows the p-PERK band-shift that results from the increased molecular weight caused by autophosphorylation [9]. As predicted, p-PERK form was only seen in Vx-809 cells treated with TG, an ER stressor, and not in cells that were left untreated or treated. Moreover, p-PERK band-shift was decreased by Vx-809 therapy even after just one hour of TG pre-treatment.

### 3.2. “Corrector” Vx-809 Interferes on Calcium Signaling

**Figure 3:**
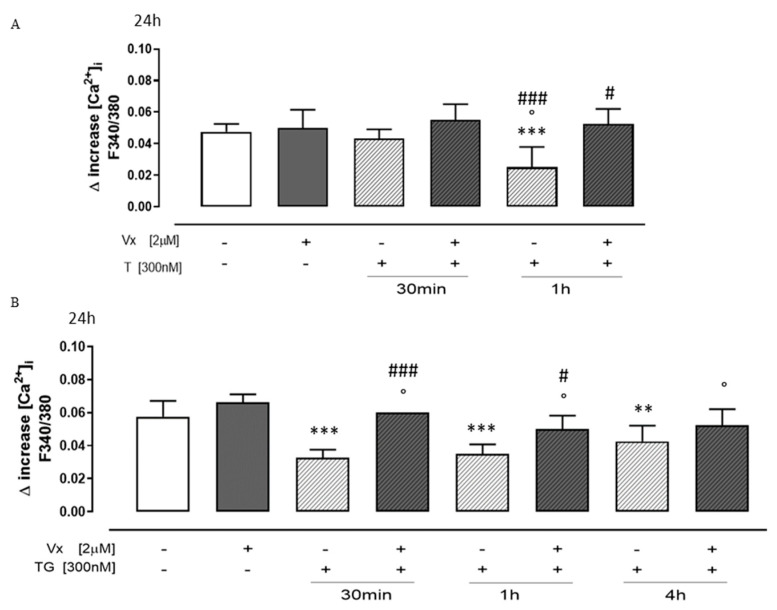
Vx-809 hampers calcium signaling. Cells were pretreated with 300 nM TG for 30 min or 1 or 4 h, to induce ER stress. Following this, Vx-809 (2 µM) was added for 24 h. After ER stress, the Vx-809 effect was determined on the reticular calcium pool in cells in calcium-free media in the presence of 1 nM TG (panel (**A**)), and intracellular calcium concentration was quantified using 1 μM of ionomycin (panel (**B**)). The results show the mean ± S.E.M. of the delta (δ) increase in the fluorescence of the FURA 2 ratio (340/380 nm) from a minimum of three separate experiments, each carried out in duplicate. The findings are presented as the average ± standard error of duplicate data from a minimum of three separate and identical tests. The Mann–Whitney U test analysis was performed on the data. ** *p* < 0.005 and *** *p* < 0.001 vs. untreated cells; ° *p* < 0.05 vs. Vx-809-treated cells; # *p* < 0.05 and ### *p* < 0.001 vs. TG-treated cells.

Misfolded proteins causing AD, PD, and prion diseases cause increases in Ca^2+^ influx and ER Ca^2+^ dyshomeostasis, resulting in excitotoxicity and ER stress in affected neurons [32]. Since impaired calcium homeostasis has been implicated in various neurological disorders, we analyzed Vx-809 treatment effects on intracellular calcium concentrations by FURA 2-AM in a calcium-free incubation medium. TG (1 nM) was used to study reticular calcium content. As shown in Figure 3A, the delta increase in reticular calcium levels in Vx-809-treated cells previously pretreated with TG at 30 min and 1 h was significantly higher (*p* < 0.05) than that in TG-treated cells, indicating higher levels of calcium stored in the ER, improving calcium homeostasis.

Ionomycin (1 μM), a potent and selective Ca^2+^ ionophore, was used to study cytosolic calcium concentration. Our results show that, in Vx-809-treated cells previously pretreated with TG, the delta increase in intracellular calcium levels was higher than that in TG-treated cells, hypothesizing an improvement in intracellular calcium storage (Figure 3B) induced by treatment with a corrector.

### 3.3. Vx-809 Counteract Thapsigargin-Induced Oxidative Stress

Several NDs may be the result of a biochemical alteration, due to oxidative stress, in bimolecular components. Moreover, the neuronal membranes exhibit a high level of polyunsaturated fatty acids, which are highly susceptible to ROS [33].

**Figure 4:**
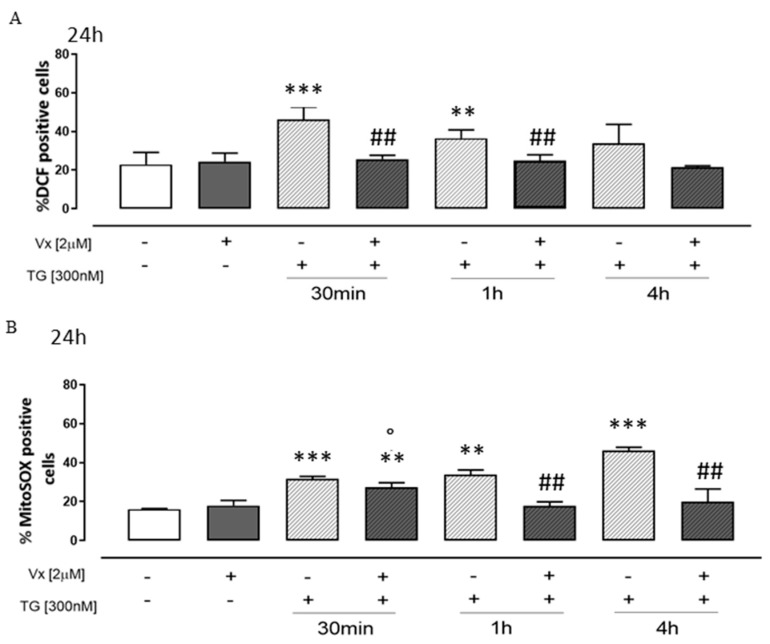

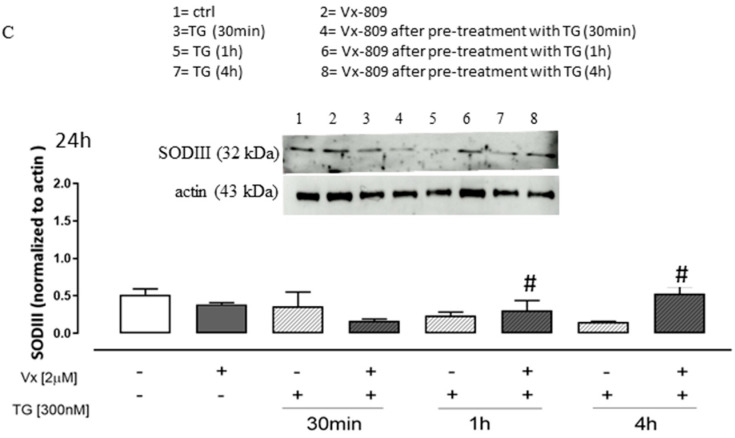
Vx-809 mitigates the oxidative damage caused by Thapsigargin. To cause ER stress, cells were pretreated with 300 nM TG for 30 min, 1 h, or 4 h. The corrector Vx-809 (2 µM) was then administered for a whole day. The probe 2′,7′-dichlorofluorescein diacetate (H_2_DCF-DA) was used to measure the amount of ROS produced in SH-SY5Y cells (Panel (**A**)). The percentage of DCF-positive cells in at least three independent experiments, each conducted in duplicate, was used to express the mean ± SEM of ROS generation. Using the MitoSOX Red probe, flow cytometry analysis was used to measure the amount of superoxide produced by the mitochondria in cells (Panel (**B**)). The expression of mitochondrial superoxide generation was calculated as the mean ± standard error of the proportion of cells positive for MitoSOX in three separate tests, each carried out in duplicate. SODIII expressions (Panel (**C**)) on neuroblastoma cells were detected by a Western blotting assay. Actin protein expression was used as loading control. The Mann–Whitney U test was used to evaluate the data. ** *p* < 0.005 and *** *p* < 0.001 vs. untreated cells; ° *p* < 0.05 vs. Vx-809-treated cells; # *p* < 0.05 and ## *p* < 0.005 vs. TG-treated cells.

To study the effects of Vx-809 on TG-induced cytosolic and mitochondrial ROS production, cytofluorimetric analyses were performed by fluorescent probe DCHF-DA and MitoSOX red, respectively. Our experiments show that Vx-809 treatment significantly decreased (*p* < 0.001) cytosolic (Figure 4A) and mitochondrial (Figure 4B) ROS production in TG-treated cells, at all experimental time points.

SODIII is the main body’s antioxidant, capable of inhibiting active oxygen damage to the organism and repairing damage caused by free radicals. Moreover, SODIII is the main antioxidant enzyme that removes superoxide anions in cells, protecting one from brain injury [34]. Western blotting analysis showed a significant increase (*p* < 0.05) in SOD III expression at 1 and 4 h from treatment in Vx-809-treated cells after induction of reticular stress compared with TG-treated cells, as depicted in Figure 4C, probably to counteract the stress induced by the increase in ROS.

### 3.4. Vx-809 Interferes with the Apoptotic Pathway

Current studies have shown that the disruption of calcium homeostasis in ER initiates early signals of apoptosis [35,36]. Apoptosis appears to play a key role in the progression of several neurologic disorders, as demonstrated by studies in animal models and cell lines [37,38]. Thus, we evaluated the percentage of hypodiploid nuclei and caspase 4 activation.

**Figure 5:**
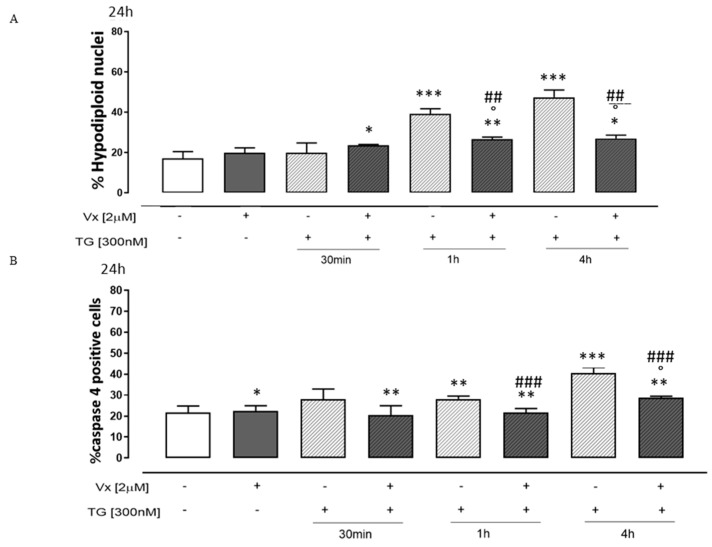
Vx-809 alters the process of apoptosis. Neuroblastoma cells were pretreated with 300 nM TG for 30 min, 2 h, or 4 h, to induce ER stress. Subsequently, 2 µM of the Vx-809 was administered for 24 h. After, the cells were stained by propidium iodide, and the fluorescence of individual nuclei was evaluated by flow cytometry (Panel (**A**)). The data are presented as the mean ± standard error of the percentage of hypodiploid nuclei from a minimum of three independent tests, each carried out in duplicate. The percentage of caspase 4 positive cells (Panel (**B**)) from at least three separate experiments, each carried out in duplicate, was given as mean ± S.E.M. The Mann–Whitney U test was utilized for data analysis. * *p* < 0.05, ** *p* < 0.05 and *** *p* < 0.001 vs. non-treated cells; ° *p* < 0.05 vs. Vx-809-treated cells; ## *p* < 0.005 and ### *p* < 0.001 vs. TG-treated cells.

In our experimental model, we show that Vx-809 administration induced a significant decrease (*p* < 0.001) in the percentage of hypodiploid nuclei in TG-pretreated SH-SY5Y cells (Figure 5A).

Caspase-4 is cleaved by calpain during ER stress, and suppression of caspase 4 activity partially inhibits cell death in humans. Therefore, caspase 4 plays a critical role in ER stress [39].

Flow cytometry analysis revealed a significant reduction in caspase 4 levels (*p* < 0.001) in treated neuroblastoma cells (Figure 5B) in ER stress conditions, suggesting that Vx-809 restores reticular homeostasis.

## 4. Discussion

Neurodegenerative diseases such as AD, PD, Huntington’s disease (HD), amyotrophic lateral sclerosis (ALS), and prion diseases (PrDs) have been found to share common cellular and molecular mechanisms including protein aggregation and formation of inclusion bodies. The initiation of misfolding in a particular cell may be a stochastic event, with constant risk throughout the individual’s life [40]. Moreover, neurons lack the ability to remove or dilute toxic molecules by mitotic division since they are terminal, highly differentiated cells; therefore, they are very sensitive to misfolded proteins to an increasing extent with aging [41].

The broad tubular–reticular system in mammalian cells, referred to as the ER, serves as a gatekeeper for the synthesis and folding of transmembrane and secreted proteins and is essential for preserving the balance of calcium, cholesterol, and lipid synthesis. ER stress can be caused by physiological, pathological, and environmental factors that interfere with the folding of ER proteins [42].

A variety of factors that obstruct the ER’s oxidative protein folding processes can cause ER stress and the buildup of intralumenal misfolded proteins [43]. When these proteins build up in the ER lumen, the UPR reaction is triggered, which can either lead to the restoration of protein homeostasis or, in the event of irreversible stress, cause cell death. A growing body of research indicates that AD-related hyperphosphorylated tau protein or Aβ peptides gradually accumulate or aggregate and cause irreversible ER stress, which in turn results in synaptic dysfunction and neurodegeneration [44]. As a matter of fact, protein misfolding-related neurodegenerative disorders are linked to the pathogenicity of chronic ER stress [43]. CFTR modulators, like Lumacaftor (Vx-809), are small molecules that bind to defective CFTR proteins and partially restore their function. Vx-809 was the first drug used in the treatment of clinical cystic fibrosis [45].

In this study, we evaluated an off-label Vx-809 use, hypothesizing that probably it does not interact directly with the mutant CFTR protein, responsible for cystic fibrosis, but with a yet unknown mechanism that acts indirectly on misfolded proteins, thus preventing the triggering of mechanisms involved in ER stress.

TG, is an experimental tool, commonly used as ER stressor, that by disturbing intracellular calcium homeostasis causes an accumulation of unfolded or misfolded proteins in the ER [9,46].

To demonstrate the neuroprotective mechanism of corrector Vx-809 on TG-induced ER stress in SH-SY5Y cells, we performed a Western blotting analysis showing that the corrector determines a significant reduction in GRP78/BiP, ATF4, ATF6, and pPERK expression, the main proteins involved in the UPR pathway, probably acting on restoring a stress-induced reticular physiological condition. Of note, our data demonstrate that the IRE1/XBP1 pathway of the UPR is blocked in Vx-809-treated cells, previously pretreated with TG. Thus, the transcription factor sXBP1 is not expressed in these cells.

Calcium is an intracellular messenger chemical found in neurons that is involved in many different activities, such as protein synthesis and energy metabolism [47,48]. The primary intracellular calcium reserve is found in the ER, which has luminal Ca^2+^ concentrations of 100–800 μM as opposed to the cytosol’s 100 nM Ca^2+^. Intraluminal chaperones, such as calnexin, calreticulin, and BiP, act as buffers by binding directly to calcium. Luminal calcium is essential for protein folding and maturation, as it binds to folding chaperones of ER and is necessary for their activity [47]. Alterations in ER calcium homeostasis are common pathological events observed in PrD models [43,48] and probably in other neuropathologies. Perturbation in calcium homeostasis directly correlated to the occurrence of ER stress and increased susceptibility to protein folding stress. Our results demonstrate, in our experimental model, that the proper restoration of protein folding, obtained by Vx-809 treatment, results in improved calcium homeostasis, by restoring the balance of reticular and cytosolic calcium, thereby reducing the free Ca^2+^ levels in neuronal cells.

The brain needs a lot of energy to function because it is one of the body’s most active organs. However, as a substrate for the production of reactive oxygen species (ROS), which are essential in physiological communication systems associated with synaptic plasticity, cell–cell connections, development of memory, cell proliferation, and apoptotic processes, oxygen molecules can seriously harm the brain under a number of circumstances (e.g., ischemia reperfusion and misfolding proteins).

Misfolded/unfolded protein aggregation causes excessive oxidative stress, endogenous reticulum stress, and mitochondrial dysfunction in neurons. However, more ER stress increases ROS production, which intensifies oxidative stress [49]. The scientific literature is replete with evidence reflecting the role of ROS in the initiation and progression of several pathologies from cardiovascular diseases to neurodegenerative diseases [4,33,50]. In this study, we have shown that Vx-809 administration, in a reticular stress model, causes a decrease in intracellular and mitochondrial ROS levels and an increase in the antioxidant enzyme SODIII, mainly at 1 and 4 h from treatment, improving the cellular redox state.

UPR failure leads to the accumulation of protein aggregates that likely cause the activation of proapoptotic caspases and induce increased apoptosis of neurons [51]. ER stress-related apoptosis could be caused by increased ROS amounts and by caspase-4 cleavage, an ER-resident caspase known to be involved in ER stress-induced apoptosis [20,24]. Here, we have established that Vx-809 treatment affects protein folding, slowing down the apoptotic process. Furthermore, cytofluorimetric analysis demonstrated a decrease in caspase 4 levels in our experimental model, given by the reduction in ER stress.

In conclusion, we can state that the Vx-809 corrector decreases UPR activation, due to ER stress conditions in neuroblastoma cells. These findings are very promising, because neurodegenerative diseases, such as AD, PD, and ALS, have an etiopathogenesis based on the accumulation of protein aggregates causing chronic reticular stress, leading neuronal cells to apoptosis or loss of function. Vx-809 activity could, therefore, reverse this trend by repairing cellular damage and restoring homeostasis within neurons, making this drug, de facto, a valuable tool in the treatment of neurodegenerative diseases.

## Data Availability

The authors confirm that the data supporting the findings of this study are available within this article.

## Abbreviations

AD	Alzheimer’s disease
ER	Endoplasmic reticulum
UPR	Unfolded protein response
IRE1α	Inositol-requiring enzyme 1 α
PERK	Protein kinase RNA-like ER kinase
ATF6	Activating transcription Factor 6
GRP78/BiP	Glucose-regulated protein 78/binding immunoglobulin protein
eIF2α	Eukaryotic translation initiation factor 2A
ATF4	Activating transcription factor 4
CHOP	C/EBP homologous protein
ERAD	ER-associated degradation
Vx-809	Lumacaftor
CF	Cystic fibrosis
NBD1	Nucleotide-binding domain 1
TMD1	Transmembrane domain 1
TG	Thapsygargin
NDs	Neurodegenerative diseases
ROS	Reactive oxygen species
PD	Parkinson’s disease
PrD	Prion disease
Xbp1	X-box binding protein 1
Xbp1s	Spliced Xbp1
CFTR	Cystic fibrosis transmembrane conductance regulator
SODIII	Superoxide dismutase III
DMEM	Dulbecco’s modified Eagle’s Medium
FBS	Fetal bovine serum
PBS	Phosphate-buffered saline
MTT	3-[4,5-dimetiltiazol]-2,5-diphenyl-2H-tetrazolium bromide
Sp	Staurosporine
ECL	Enhanced chemiluminescence
HBSS	Hank’s balanced salt solution
H_2_DCF-DA	2′,7′-dichlorofluorescin diacetate
MitoSOX	Mitochondrial superoxide
PI	Propidium iodide
SERCA	Sarco/endoplasmic reticulum Ca^2+^-ATPase
ALSIDP	Amyotrophic lateral sclerosisIntrinsically disordered proteins
